# Predicting Drug-Induced Liver Injury Using Machine Learning on a Diverse Set of Predictors

**DOI:** 10.3389/fphar.2021.648805

**Published:** 2021-08-18

**Authors:** Temidayo Adeluwa, Brett A. McGregor, Kai Guo, Junguk Hur

**Affiliations:** ^1^ Department of Biomedical Sciences, University of North Dakota, Grand Forks, ND, United States; ^2^ Department of Neurology, University of Michigan, Ann Arbor, MI, United States

**Keywords:** DILI, Connectivity Map, Tox21, FAERS, machine learning, Mold2

## Abstract

A major challenge in drug development is safety and toxicity concerns due to drug side effects. One such side effect, drug-induced liver injury (DILI), is considered a primary factor in regulatory clearance. The Critical Assessment of Massive Data Analysis (CAMDA) 2020 CMap Drug Safety Challenge goal was to develop prediction models based on gene perturbation of six preselected cell-lines (CMap L1000), extended structural information (MOLD2), toxicity data (TOX21), and FDA reporting of adverse events (FAERS). Four types of DILI classes were targeted, including two clinically relevant scores and two control classifications, designed by the CAMDA organizers. The L1000 gene expression data had variable drug coverage across cell lines with only 247 out of 617 drugs in the study measured in all six cell types. We addressed this coverage issue by using Kru-Bor ranked merging to generate a singular drug expression signature across all six cell lines. These merged signatures were then narrowed down to the top and bottom 100, 250, 500, or 1,000 genes most perturbed by drug treatment. These signatures were subject to feature selection using Fisher’s exact test to identify genes predictive of DILI status. Models based solely on expression signatures had varying results for clinical DILI subtypes with an accuracy ranging from 0.49 to 0.67 and Matthews Correlation Coefficient (MCC) values ranging from -0.03 to 0.1. Models built using FAERS, MOLD2, and TOX21 also had similar results in predicting clinical DILI scores with accuracy ranging from 0.56 to 0.67 with MCC scores ranging from 0.12 to 0.36. To incorporate these various data types with expression-based models, we utilized soft, hard, and weighted ensemble voting methods using the top three performing models for each DILI classification. These voting models achieved a balanced accuracy up to 0.54 and 0.60 for the clinically relevant DILI subtypes. Overall, from our experiment, traditional machine learning approaches may not be optimal as a classification method for the current data.

## Introduction

Adverse drug reactions (ADRs) are a common concern of novel drugs and therapeutics. One of the more common targets of ADRs is the liver due to its role in the metabolism of compounds and resulting liver damage is termed as Drug-Induced Liver Injury (DILI) (Daly, 2013; Atienzar et al., 2016; Marzano et al., 2016). DILI is a unique challenge in drug development due to the inability of animal models to translate to human clinical trials in treatment populations. Assessing DILI risk has been approached in multiple ways during drug development; however, officials often rely on post-marketing surveillance to detect possible long-term side effects such as DILI (Berlin et al., 2008). The U.S. Food and Drug Administration (2021) has established the DILIrank dataset, the largest reference drug list ranked for DILI risk in humans, to facilitate the development of predictive models by enhancing drug label DILI annotation with weighted causal evidence (Chen et al., 2016b). This dataset contains four classifications, including most, less, ambiguous, and no-DILI concern, regarding 1,036 FDA-approved drugs. Additionally, predicting DILI is difficult due to the absence of specific and reliable biomarkers. Traditional biomarkers, including alanine aminotransferase, total bilirubin levels, aspartate aminotransferase, and gamma-glutamyl transferase (among others) are not specific enough to separate DILI from other forms of liver injury (Ozer et al., 2008). Due to this reason, FDA in 2016 approved investigations into glutamate dehydrogenase and microRNA-122 as potential biomarkers (Andrade et al., 2019; López-Longarela et al., 2020). Messner and others characterized exosomal microRNA-122 in methotrexate and acetaminophen-induced toxicity in hepatic stem cells, HepaRG. They confirmed that microRNA-122 can be used as a sensitive biomarker for DILI (Messner et al., 2020).

Predictive markers of DILI, determined by compound properties and known variables rather than preclinical studies, would facilitate drug development in a wide variety of ways (García-Cortés et al., 2018; Saini et al., 2018). Multiple groups have attempted to predict DILI using drug compounds or proposed drug properties. Chemical structures (Shin et al., 2020), gene expression response (Liu et al., 2020), and patient genetic data have been previously used for DILI prediction using traditional machine learning algorithms. Xu et al. proposed a deep learning model built on a “combined data set” gathered from a variety of sources and used a molecular structural encoding approach for the chemical structures of the drugs in their data (Xu et al., 2015). Kohonen et al. proposed a “big data compacting and data fusion” concept (Kohonen et al., 2017). In their approach, the authors utilized data from the Connectivity Map (CMap; Broad Institute) database, the Open Toxicogenomics Project-Genomics Assisted Toxicity Evaluation Systems (TG-GATEs; National Institutes of Biomedical Innovation, Japan), the US National Cancer Institute 60 tumor cell line screening (NCI-60), and the US FDA Liver Toxicity Knowledge Base (LTKB). Using these databases, they modeled a predictive toxicogenomics space that captured all possible well-known hepato-pathological changes (Kohonen et al., 2017).

Building upon these previous efforts to accurately predict DILI, the Critical Assessment of Massive Data Analysis (CAMDA) in collaboration with the Intelligent Systems for Molecular Biology (ISMB) has proposed the CMap Drug Safety Challenge for their annual conferences in 2018, 2019, and 2020 (Table 1). The previous challenges in 2018 and 2019, while sharing a similar goal to predict potential liver toxicity, also had distinct parameters. The prediction DILI classification in 2018 was a binary positive or negative DILI status, while in 2019 the challenge was more focused on the potential DILI risk ranging from no concern to most concern with four classifications reflecting the DILIrank dataset (Chen et al., 2016b). The data, used for predicting the DILI classification of drugs in the 2018 challenge, were limited to microarray data from MCF7 and PC3 cell lines. Chierici et al., in 2018 employed deep learning techniques for the microarray data from 276 compounds but only achieved Matthews Correlation Coefficient (MCC) values of <0.2 (Chierici et al., 2020). Sumsion et al. in the same challenge year utilized more traditional classification algorithms along with soft voting but reached a maximum MCC of 0.2 and maximum accuracy of 70%, while the voting model never performed the best when compared to individual models (Sumsion et al., 2020). Both studies cite struggles with the small sample size and imbalanced datasets; however, resampling, in this case, led to overfitting rather than improved testing accuracy.

**TABLE 1:** Previous CAMDA Drug Safety Challenge Summary. The CMap Drug Safety Challenge has been a repeated effort by CAMDA to develop predictive models for DILI. Previous studies are cited by their year of publication and leading author while also describing the year in which the challenge was administered by CAMDA and relevant data sources and DILI classifications for prediction.

Authors	CAMDA drug safety challenge	Data sources	DILI conditions
Current: Adeluwa et al.	2020	CMap L1000, MOLD2, FAERS, TOX21	DILI1, DILI3, DILI5, DILI6
2021: Liu et al.	2019	CMap L1000, SMILES strings, SIDER 4.1	Most-DILI concern, Less-DILI concern, ambiguous DILI concern, No-DILI concern
2021: Aguirre-Plans et al.	2019	CMap L1000, DisGeNET, GUILDify, SMILES, DGldb, HitPick, SEA	
2021: Lesinski et al.	2019	CMap L1000, SMILES, annotated images	
2020: Chierici et al.	2018	Affymetrix GeneChip (MCF7, PC3)	DILI-1, DILI-0
2020: Sumsion et al.	2018	Affymetrix GeneChip (MCF7, PC3)	

The CMap Drug Safety Challenge expanded in 2019 by including not only expression data from L1000 CMap but also by allowing a wide variety of external data sources that were incorporated into each study. Lesinski et al. achieved their best predictive results by incorporating molecular drug properties along with the most informative variables from five of 13 cell line expression models *via* a super learner method (Lesiński et al., 2021). Including molecular property information improved their cell line models’ accuracy up to 73% utilizing a random forest algorithm, which originally ranged from 55 to 61%. Liu et al. built support vector machine and random forest models using chemical descriptions from DILIrank annotation along with expression values from predicted protein targets (Liu et al., 2021). This approach produced models with an accuracy of 75.9% that were also able to correctly identify targets associated with the mechanism of action and toxicity of nonsteroidal anti-inflammatory drugs, a class of drugs commonly associated with DILI. Aguirre et al. utilized the widest array of predictive data, including L1000 CMap expression, drug-target associations, structural data, phenotype-associated gene signatures, protein-protein interactions, and drug targets data (Aguirre-Plans et al., 2021). Their models’ accuracy remained comparable to other study results at 70%, but they also identified structural dissimilarities within the DILI risk labels used. All three published studies from the 2019 CMap drug safety challenge cited data limitations within their study, including complex dosage-related toxicity, a small sample size, and a small number of compounds with hepatoxicity annotation.

The current CAMDA 2020 challenge was structured in a way to address the previous limitations, while also redefining the relevant DILI classifications. The challenge aimed to predict or classify positive and negative classes within each of four DILI designations, namely DILI1, DILI3, DILI5, and DILI6. DILI1 and DILI3 were clinical classifications based on specific severity scores or established FDA warnings and precautions, while DILI5 and DILI6 served as a negative and positive control class, respectively (Table 2). Drug class labels were assigned by the CAMDA 2020 challenge organizers. DILI1 was described as a severity score ≥, six which is associated with high risk based on the DILIrank dataset and LTKB (Chen et al., 2016a). DILI3 was described as drugs withdrawn, given boxed warnings, or warnings and precautions from the FDA due to either known risk factors or adverse event reporting. DILI5 served as a randomly assigned negative control, while DILI6 was constructed as a positive control based on molecular weight with positive compounds weighing >320 g/mol. The drug list for the study was expanded to 617 drug compounds to improve on the sample size limitations of previous studies; however, these datasets remained highly imbalanced.

**TABLE 2:** Drug-Induced Liver Injury Classifications. Four binary classes of DILI were provided by the CAMDA organizers. DILI1 positive compounds were based on the clinical severity score associated with liver necrosis. DILI3 positive compounds were based on drugs already associated with warnings and precautions or that have been withdrawn due to liver toxicity. DILI5 was a random assignment from the organizers as a negative control group while the DILI6 classification was based on molecular weight (>320 g/mol) to serve as a positive control.

Targets	Positive group	Negative group
DILI1	DILI severity score ≥6 (N = 141)	DILI severity score <6 (N = 476)
DILI3	Withdrawn, box warning, warning and precaution (N = 227)	Adverse events and no match (N = 390)
DILI5	Assigned DILI endpoint 1 (N = 308 positive)	(N = 309 negative)
DILI6	Assigned DILI endpoint 2 (N = 318 positive)	(N = 299 negative)

Note1: DILI5/DILI6 are controls; DILI5 is randomly split; DILI6 is the positive control, dividing compounds based on their molecular weight > 320 g/mol.

The imbalance within the clinically relevant DILI data is expected considering that many approved drugs do not have a significant hepatoxicity risk; however, the control classes of DILI5 and DILI6 were structured in a balanced manner (Table 3). For this challenge, L1000 drug expression signatures from primary human hepatocytes (PHH), liver carcinoma (HepG2), immortalized kidney cells (HA1E), human skin melanoma (A-375), breast cancer (MCF7), and adenocarcinoma (PC-3) were used as inferred from landmark genes defined by Connectivity Map (Subramanian et al., 2017). These expression responses were simplified to one specific dose at one specific treatment time in order to yield the largest available dataset for training and testing while also addressing previous dosage toxicity concerns. Other non-gene expression data provided included molecular descriptors encoding two-dimensional chemical structure information from MOLD2 (Hong et al., 2008), post-marketing drug adverse event information from FAERS (FDA adverse event reporting system (FAERS), 2015), and high-throughput liver toxicity screening results from TOX21 (Huang et al., 2016). While previous studies also utilized external data sources to improve model performance, the current study focuses on the various types of data processed and provided from the CMap drug safety challenge.

**TABLE 3:** Training Data Imbalance. The data used for the clinical DILI classes of DILI1 and DILI3 were imbalanced which negatively influenced the models built to predict these classes.

DILI class	Negative	Positive
DILI1	326	96
DILI3	262	160
DILI5	218	204
DILI6	197	225

We constructed models to predict each drug’s DILI class (positive or negative) within the four DILI classifications (DILI1, DILI3, DILI5, and DILI6) by first evaluating the performance of each dataset in predicting DILI and also by employing ensemble voting with the top three performing models across data types. The gene expression data presented a unique challenge in that not all drugs were tested in each cell line or even in liver-relevant cell lines. To address this, we utilized a Kru-Bor merging method to merge the expression signatures across cell lines into one representative drug signature (Iorio et al., 2010; Lin, 2010). These expression signatures were narrowed down to the top and bottom 100, 250, 500, and 1,000 ranked genes and subjected to feature selection *via* a Fisher’s exact test based on their involvement in DILI positive/negative assigned drugs for each DILI class. FAERS, MOLD2, and TOX21 datasets were also used to construct DILI predictive models, and to address the imbalance of these data we tested resampling techniques. Various traditional classifier algorithms were used to build models on these datasets, and the models were evaluated on a blinded test set by the CAMDA committee. Based on the training area under the curve (AUC) values of these models, the top three algorithms for each datatype (cell expression, FAERS, MOLD2, and TOX21) for each DILI class were included in our ensemble voting model. We tested hard, soft, and weighted voting across these datasets to see if the varying dimensions of data can improve predictive performance.

## Materials and Methods

### Data Processing

**FIGURE 1:**
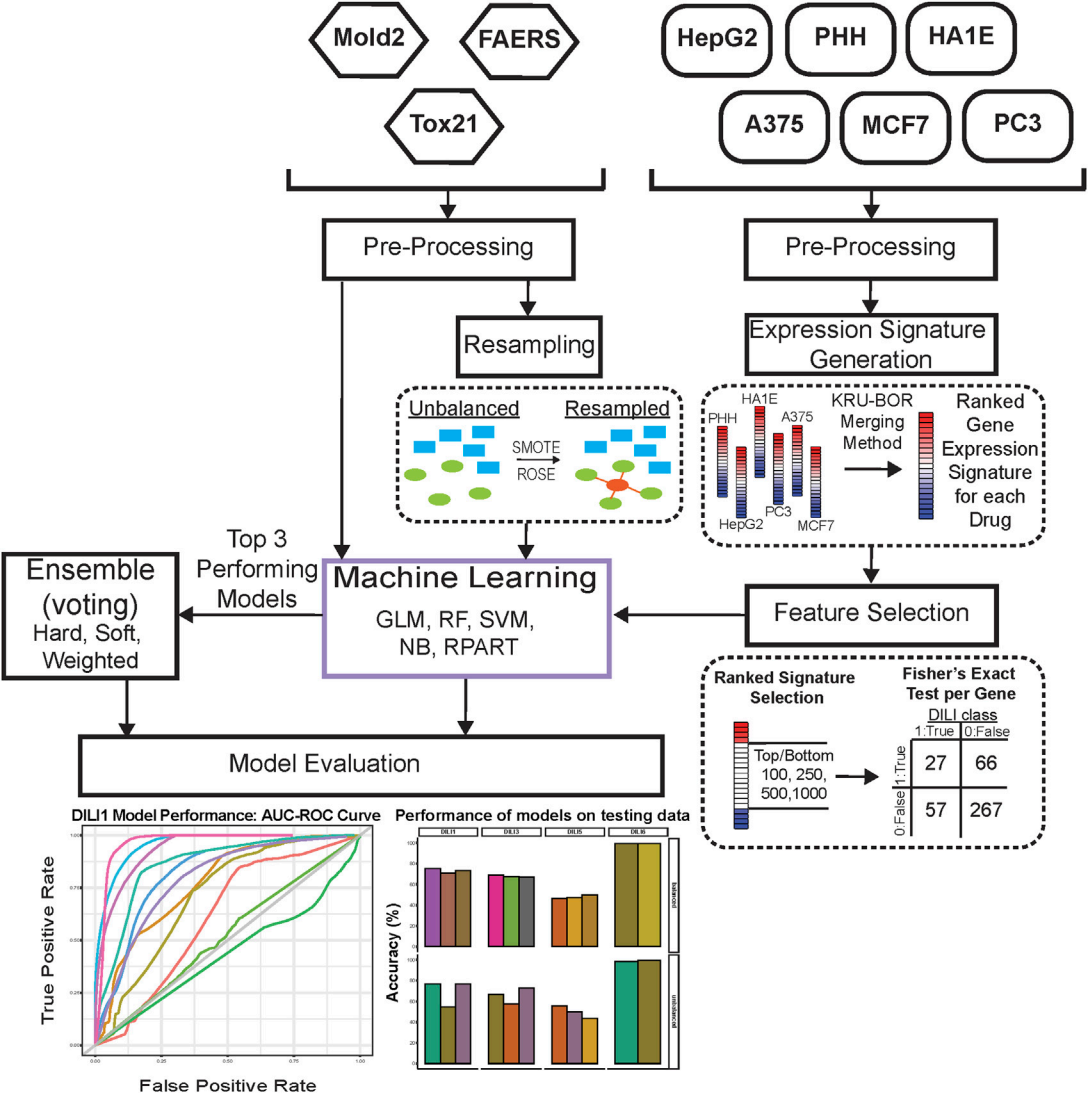
Study Workflow. Data were separated into expression-based datasets and non-expression-based (FAERS, MOLD2, TOX21) for testing. Non-expression data was evaluated with resampling methods ROSE and SMOTE as well as an unbalanced dataset. Expression-based datasets were merged across cell lines into one representative expression signature per drug. These signatures were tested as the top and bottom 100, 250, 500, and 1,000 ranked genes for each drug. Following signature formation, feature selection using a fisher’s exact test was used to determine significant predictors of DILI classification. Machine learning was used on predictors for both expression-based and non-expression models, which were evaluated based on training AUC curve values as well as testing performance. The top three performing models for each DILI type were utilized in ensemble voting models in an effort to incorporate both expression and non-expression datasets.

**FIGURE 2:**
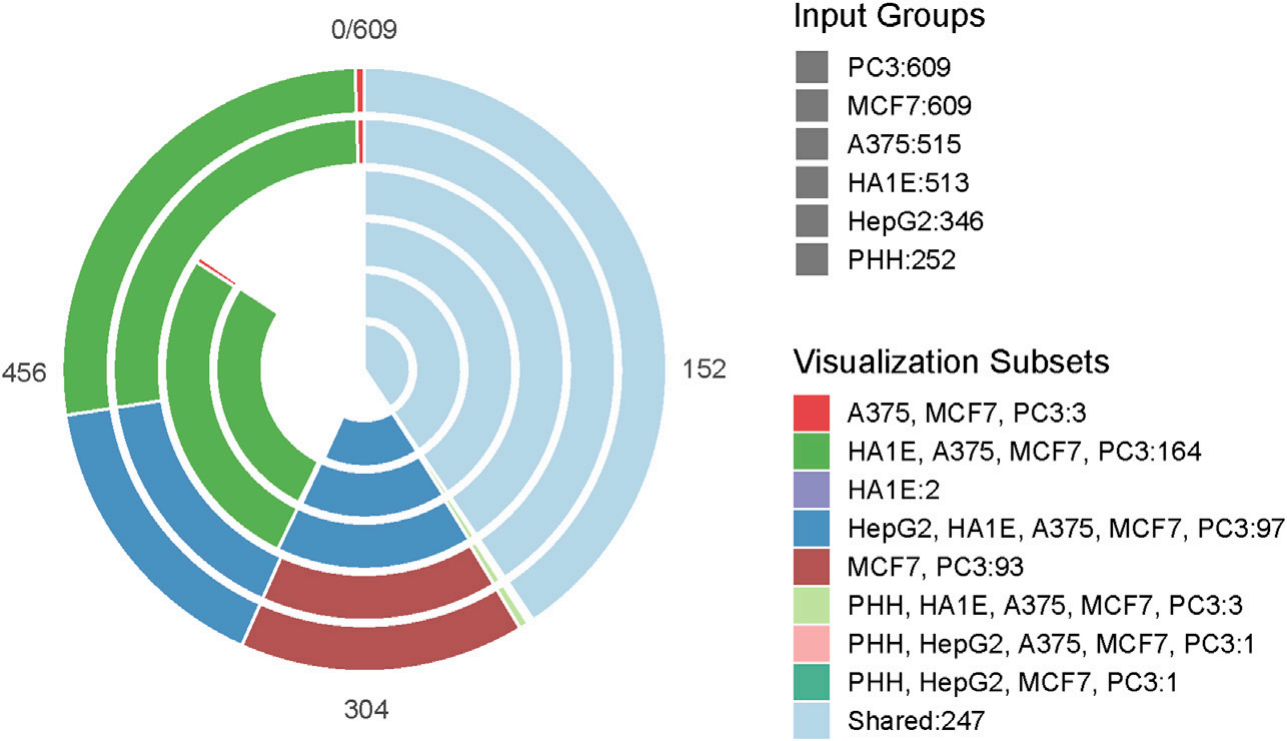
Drug Testing Cell Distribution. The Venn-Pie diagram depicts the overlap of drugs tested between each of the six cell lines used in this study. Each bar within the Venn-Pie represents an individual dataset while the color of the bars indicates the overlapping group of compounds across datasets. While 247 of the 617 drugs included in the training and test data were tested in all six cell lines, some compounds were only tested in a singular cell line and others did not have any expression information provided.

The overall workflow of our study is shown in Figure 1. Initially, the overlap of drugs, included in each of the gene expression cell data sets, was investigated using VennDetail (Guo and McGregor, 2020) to create a Venn pie chart showing the various drug testing subsets across the six cell lines (Figure 2). Each of the non-gene expression datasets (FAERS, MOLD2, and TOX21) were treated as individual datasets, while the gene expression data were merged across cell lines to build classifier models. In general, we used standard preprocessing techniques, including removing zero variance features and missing values. DILI1 and DILI3 suffered from class imbalance (Table 3). For all non-gene expression data, to mitigate this issue, we attempted three oversampling techniques, including synthetic minority oversampling technique (SMOTE) (Chawla et al., 2002), random oversampling examples (ROSE) (Menardi and Torelli, 2014), and a random upsampling of the minority classes. SMOTE balances data by randomly creating artificial samples between two nearest-neighbor samples, while ROSE uses a smoothed bootstrap technique to resampled the data (Chawla et al., 2002; Menardi and Torelli, 2014). For comparison, models were built using imbalanced data as well. Before training non-gene expression datasets, they were standardized to have a mean of zero and a standard deviation of one. Preprocessing details specific to each dataset as well as some characteristics of the data are discussed below.

### Food and Drug Administration Adverse Event Reporting System

The CAMDA organizers provided us with FAERS data for all 617 drug compounds. Of these, 422 were grouped as “training data”. This dataset contains 20 features corresponding to information on the percentage of reported adverse events for each drug compound by gender and age group demographic. After removing highly correlated features, we upsampled the data to cater to the class imbalance by randomly sampling with replacement from the minority class to balance the majority class. An additional preprocessing step was to create two new variables, namely “male ratio” and “female ratio”, taking into account all reported events irrespective of the gender, all reported DILI events irrespective of gender, and the percentage of reported DILI events by gender.

### Toxicology in the 21st Century

In addition to the FAERS dataset, we were provided with concentration-response information of 600 drugs. Of these, 412 were designated as “training data”. Thirty-two features corresponded to concentration-response curve ranks. Out of all 412 drugs for training, 57 drugs were removed for missing values. In addition, we removed highly correlated features using an arbitrary cutoff of 0.82 and catered to the class imbalance by using SMOTE.

### Molecular Descriptors from 2D Structures

Alongside the FAERS and TOX21 data provided, we had access to the 2D molecular descriptors or structural information of these 617 drug compounds. 422 of these drugs were designated for training. There were 777 features for each drug compound with each feature corresponding to MOLD2 descriptors. To cater to class imbalance, we upsampled minority classes, as well as ROSE, and SMOTE.

### Connectivity Map L1000 Gene Expression Data

The L1000 assay data used in this study is a high-throughput gene expression assay that measures mRNA transcript abundance of 978 landmark genes based on an inference algorithm to infer the expression of 11,450 additional genes in the transcriptome (Subramanian et al., 2017). Utilizing simulation, it has been observed that this reduced representation of the transcriptome can recapitulate around 80% of the relationships of measuring the entire transcriptome directly. In this study, 12,328 deidentified predictors genes were provided by the CAMDA organizers with Z scores to indicate transcript abundance. The treatment time and dosage of each drug were selected by the CAMDA committee to produce the largest available dataset for both test and training data.

### Kruskal-Borda Merging

Since not all drugs were tested in each cell line data made available, we utilized the Kruskal-Borda (Kru-Bor) merging algorithm in the GeneExpressionSignature R package (Li et al., 2013). This approach allowed us to generate a unified drug-induced expression signature across cell types since many drugs were not tested in the PHH or HepG2 liver cell lines. The Kruskal algorithm (Kruskal, 1956) finds a minimum spanning forest of an undirected edge-weighted graph while the Borda merging method (Saari, 2000b; 2000a) uses ranked options in order of preference to determine the outcome. Thus each closest neighbor in rank merges one by one until a unified signature is formed. Following merging, the top and bottom 100, 250, 500, and 1,000 ranked genes were selected as drug signatures for feature selection.

### Feature Selection

A method of feature selection utilized across the merged signatures produced *via* our Kru-Bor merging was based on a gene’s significance (*p*-value < 0.01) in predicting the DILI score *via* a Fisher’s exact test. If a gene is included in the top or bottom 100, 250, 500, or 1,000 ranked list, depending on the model data, for any drug it would be assigned a one (True), or if it fell outside of that range it would be assigned a zero (False). The classifier for each type of DILI was also one (DILI positive) or zero (DILI negative). We used these classifiers to identify if these highly influenced genes were predictive of a drug being DILI positive or DILI negative with a *p*-value cutoff of 0.01.

### Machine Learning

The prediction of DILI was treated as a binary classification problem for each DILI type. That is, for each of DILI1, DILI3, DILI5, and DILI6, outcomes were split between “positive” and “negative”. We used a 5-folds cross validation repeated 100 times, and a random search strategy to search for the best parameters for each model. The data was made available such that training and test sets had been pre-identified. Importantly, we did not have access to the correct labels for the test data. Models were built using traditional machine learning algorithms within the caret (Kuhn, 2008) package in R version 4.0.0 (R Core Team (2020), 2020).

The machine learning algorithms we used are suitable for classification tasks. They include a Logistic Regression (LR) (Cox, 1958), Linear Discriminant Analysis (LDA) (Li and Jain, 2009), Decision Trees (DT) (Quinlan, 1986), Support Vector Machines (SVM) (Cortes and Vapnik, 1995), Naïve Bayes (NB) (Hand and Yu, 2001), a One-layer Neural Network (Nnet), and a Random Forest (RF) algorithm. LR and LDA are generally categorized as linear classification models, with an assumption that the data follows a normal distribution. Given a set of predictors, LR aims to build a linear model of these predictors by minimizing the sum of squared residuals. LDA uses the prior probability of belonging to a class to estimate posterior probabilities by using Bayes’ Theorem. DT and RF are often classified as trees and rules-based algorithms. Given a set of predictors, a decision tree works by using if-else conditions to build a definitive set of rules using splits. The challenge usually lies in determining optimal situations to apply a “then”-clause (or a split). In RF, similar conditional statements are used. However, instead of using the entire sample of data for tree-building, RF uses many independent subsamples from the training data to build small decision trees. Each small decision tree classifies an observation by voting. Neural networks and SVMs are generally grouped as non-linear algorithms. Neural networks (in our case, a multilayer perceptron i.e. a neural network with one hidden layer), are modeled after how neurons in the human brain work. The outcome or prediction is a linear combination of the hidden layer(s) transformed by a non-linear activation function. There are several activation functions used, depending on whether the problem is a regression or classification problem. In our case, we used a sigmoidal or logistic function, since we were dealing with a classification problem. SVM aims to find support vectors or data points that separate the different classes as much as possible. Intuitively, these data points are the most difficult to separate (the reasoning is that they lie very close to one another and to the hyperplane or decision boundary), and are thought of to be important in separating classes. There are different flavors of SVMs depending on the kernel used (kernels are similar to non-linear activation functions used in neural networks). In the current study, we used polynomial, linear, and a radial basis function kernels.

### Model Evaluation

To evaluate the performance of our models, we focused on the area under the ROC curve (AUC) value as well as the specificity, sensitivity, accuracy, and MCC of the models on the test set. The AUC value is a widely-used metric in binary classification problems. An AUC value of one indicates a perfect classifier, i.e. a model that is perfectly able to separate both classes, while an AUC value of 0.5 indicates a model that predicts at random. Depending on the application domain, AUC values of 0.7 and above are usually acceptable. Specificity measures the ratio of negative classes that were correctly identified by the model out of all negative classes, while sensitivity measures the ratio of positive classes that were correctly identified by the model out of all positive classes. These metrics are affected by how the target labels are structured and passed to the algorithm, and they range from 0 to 1. Additionally, we evaluated the performance of our models on the test set by calculating the balanced accuracy of prediction. Balanced accuracy is the average of the sensitivity and specificity or the average of the fraction of correct labels that are predicted correctly (by the model) within each class. We used this metric because we observed that there was class imbalance within our datasets regardless of DILI type.

MCC is particularly useful in datasets of different class distributions (or imbalanced data) because it considers all of the false and true positives and negatives. It is calculated from the confusion matrix of a model and its values range from +1 to −1, with +1 indicating a perfect classification, 0 indicating random classifications, and −1 indicating no relationships between the observed and predicted classes.

### Ensemble Voting Machine Learning

In an attempt to improve the classification accuracy of our models, we used three ensemble voting approaches, namely soft voting, hard voting, and a weighted voting approach. These ensemble methods work best when there are varying algorithms of different strengths i.e. algorithms having varying underlying assumptions about the data, and when each one has reasonable predictive power (Kuncheva, 2002; Van Erp et al., 2002). Using the gene expression data provided by CAMDA 2018 organizers, Sumsion and others (Sumsion et al., 2020) used hard and soft voting ensemble methods in an attempt to improve prediction accuracy on DILI risk. As an extension of their work, we hypothesized that since we have access to larger and more diverse datasets, we could capture different aspects of predicting DILI types and use these ensemble methods to improve prediction.

Hard voting, also known as majority voting, takes into account the predicted class labels of each classifier (or voter) (Ruta and Gabrys, 2005). Voting is done by counting how many class labels (for each class) were predicted among all classes. The class label with the highest count is taken to be the predicted class label for that observation. On the other hand, soft voting considers the probabilities of each class label by each classifier (Lin et al., 2003). In other words, it considers how certain each classifier is about the class labels. For each class label, the probabilities are averaged, and the label with the highest average probability is taken as the predicted class label for the observation.

The third approach to voting involves using a weight to skew predictions towards the most certain models. In our approach, we used the AUC of each classifier as a weighting parameter for the output probabilities. This was done to take into account that some classifiers might have better predictive power and should be given preference in determining the outcome of the voting. To weigh each probability, we multiplied the probabilities of each predicted class by the AUC and divided this by one subtracted from the weight, that is, the AUC of that model. Afterward, weighted probabilities were treated just as in soft voting: by taking the average of all resulting weighted probabilities belonging to each class. The class label with the higher average was taken as the predicted class for that observation. Therefore, the predicted class, $\hat{y}$, of observation, given an output set of class membership probabilities across many models, P, is given by:

$$C\left(\hat{y}|P\right)=argmax\;\;\frac{1}{m}\sum_{i=1,2\ldots m}^{m}\left(\frac{w_{i}^{m}*p_{i}^{c}}{w_{i}^{m}-1}\right)\;$$

Where m is a model, $w_{i}^{m}$ is the weighting parameter for a model, $p_{i}^{c}$ is the probability of class membership.

## Results

### Food and Drug Administration Adverse Event Reporting System Modeling

**FIGURE 3:**
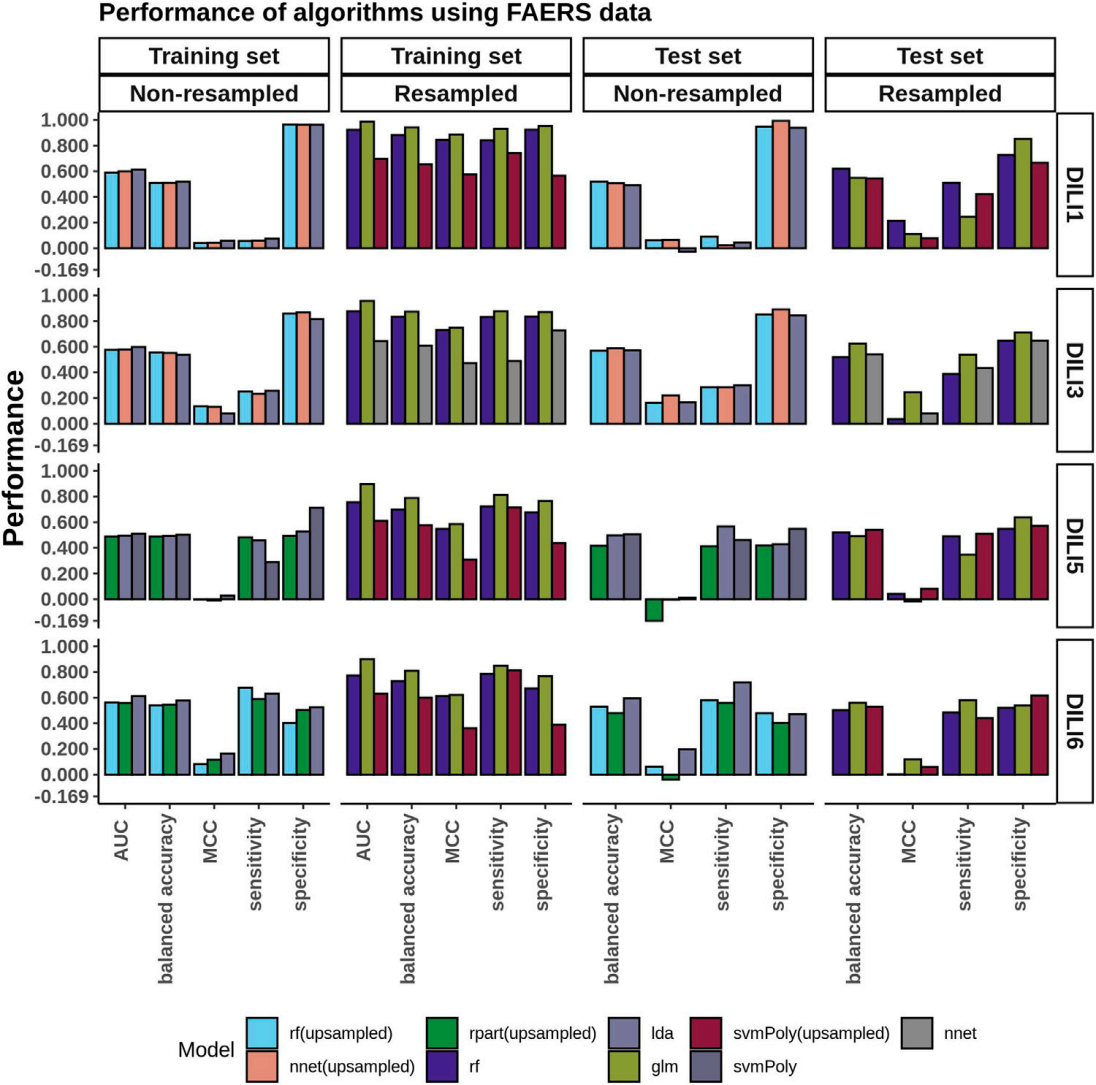
FAERS Model Performance. Performance evaluation of the DILI predictive models built using the FAERS reporting data was conducted on both the original unbalanced and the resampled/balanced datasets. The best performing algorithm determined by AUC between GLM, IDA, NB, NNET, RF, RPART, and SVMPoly were selected. For DILI1 and DILI3, the highest accuracy was 0.62 with MCC values of 0.21 and 0.24, respectively.

The performance of FAERS data in predicting each of the DILI types can be seen in the bar plots in Figure 3. While we built many models, we compared and picked the best three models based on the AUC values to predict DILI class on the test set. We noticed that using the raw data (without resampling), models achieved classification accuracy between 0.51 and 0.55 and MCC between 0.04 and 0.14 on the training set and did not do noticeably better on the test set (accuracy: 0.49 to 0.59, MCC: −0.03–0.22). On the other hand, using resampled datasets improved the accuracy of the models on the training set to a range of 0.61–0.94 (MCC: 0.47–0.89). Using these models to predict the DILI class of the test set showed a slight improvement in the accuracy (0.52–0.62). The MCC, however, was between 0.04 and 0.24.

### TOX21 Modeling

**FIGURE 4:**
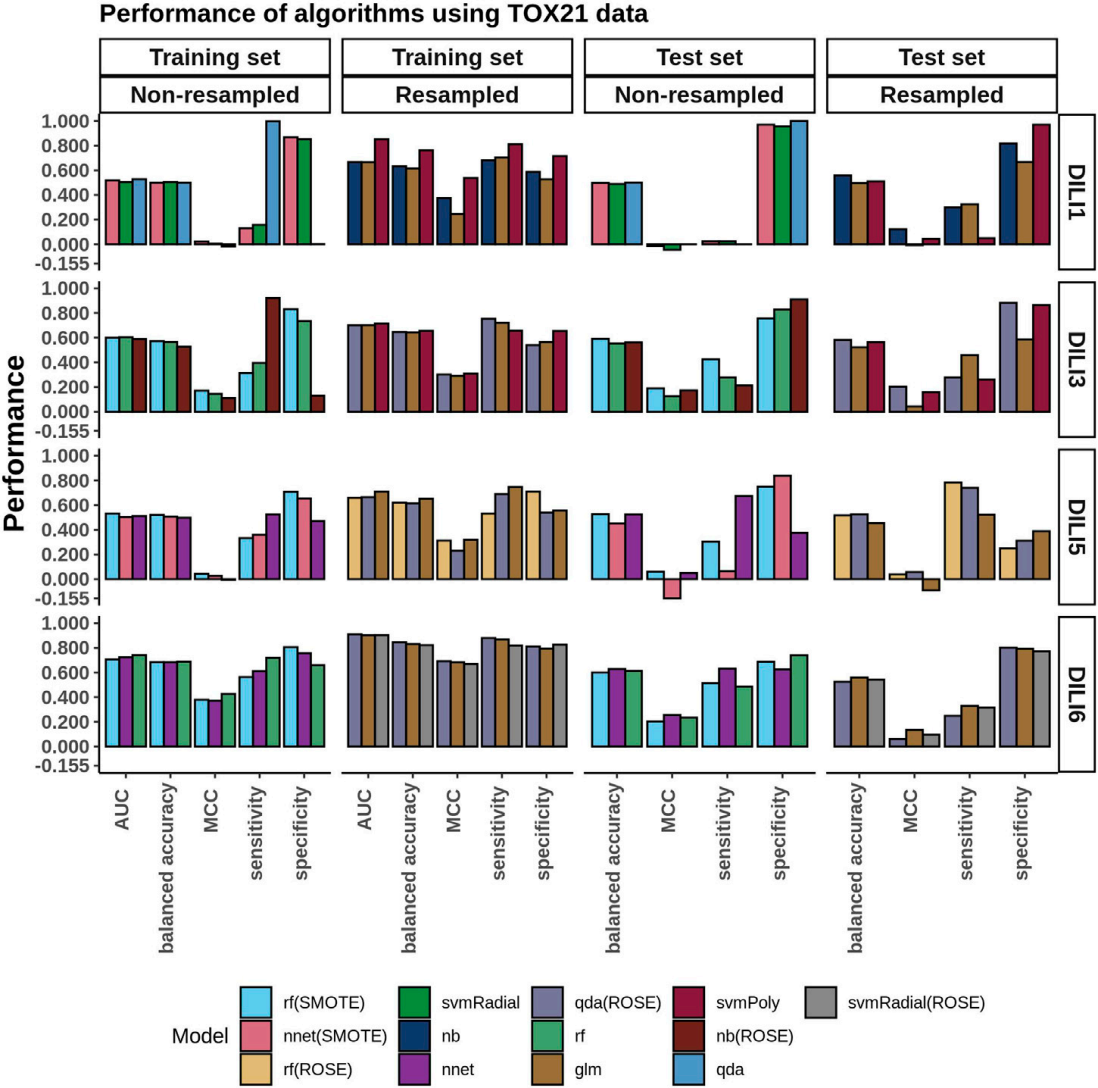
TOX21 Model Performance. The performance of DILI predictive models built using the toxicology information provided from TOX21. The three best-performing algorithms, based on training AUC and based on whether resampling was used or not, are presented in the bar plots.

The top three models built using TOX21 data (using the AUC as the criterion) were evaluated on the test set (Figure 4). Using the data as is, without resampling, the accuracy of the training data was between 0.50 and 0.57 (MCC: −0.02–0.17). As expected, the models failed to generalize to the test set (accuracy: 0.50 to 0.59, MCC: −0.04–0.19). Again, we observed that resampling slightly improved the accuracies of these models on the training set (accuracy: 0.62–0.76, MCC: 0.25–0.54). Yet, there was no major improvement on the test set (accuracy: 0.50 to 0.58, MCC: −0.01–0.20).

### MOLD2 Modeling

**FIGURE 5:**
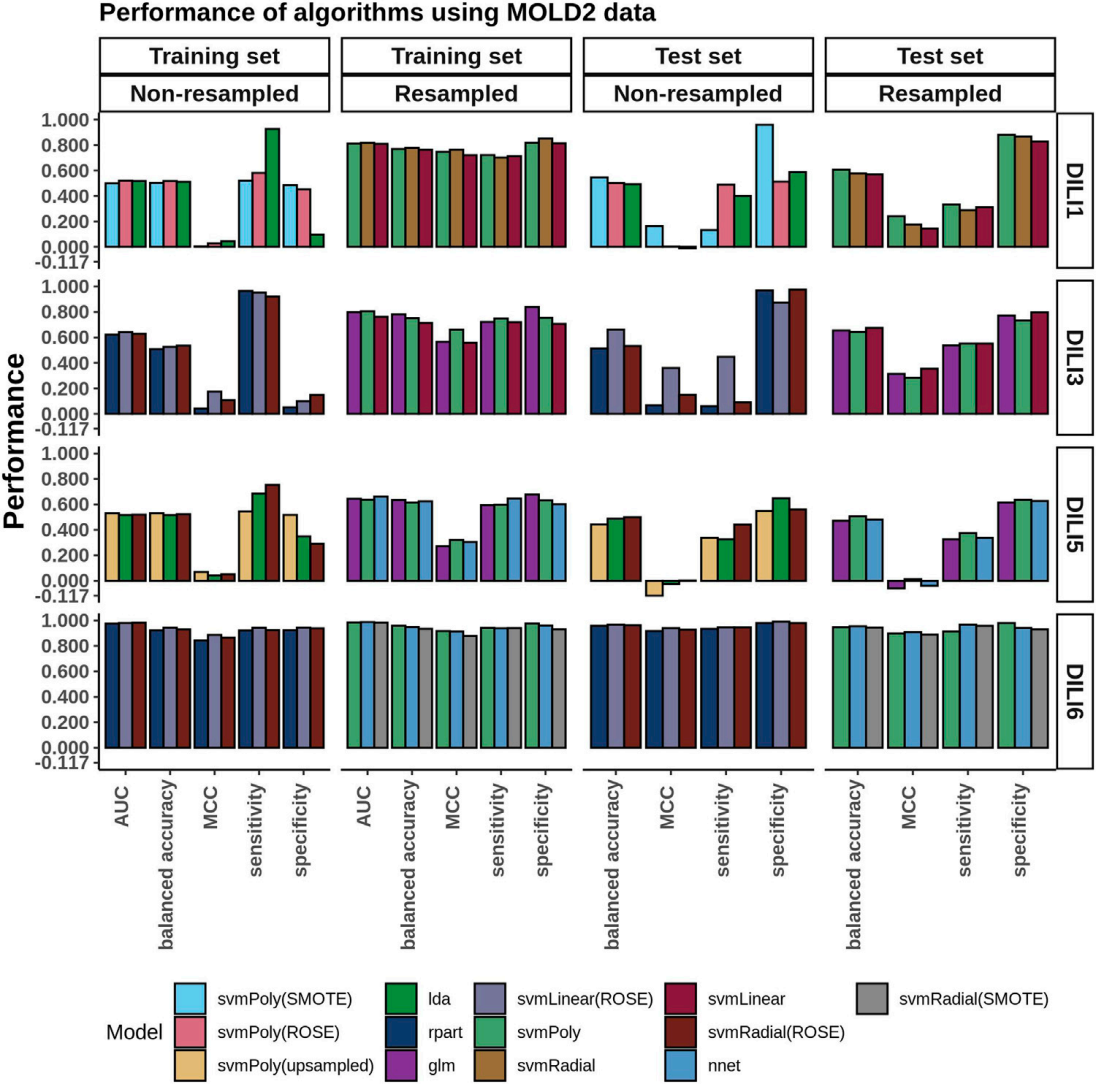
MOLD2 Model Performance. The chemical structural information from MOLD2 was imbalanced between DILI positive and negative samples. Predictive models were evaluated on both the unbalanced and resampled/balanced datasets. The three best-performing models for each DILI type, based on AUC and resampling methods, are depicted in the bar graphs.

Similarly to how the FAERS data was handled, we selected the top three performing models built using MOLD2 data in each category (resampled or non-resampled) to predict the DILI class of the test data (Figure 5). Models built using the non-resampled MOLD2 dataset gave accuracies of 0.50–0.54, showing that the models were randomly predicting the classes (MCC: 0.00–0.17). This performance was similar on the test set (accuracy: 0.50 to 0.66, MCC: −.01–0.36) with a slight improvement. Similarly to what we observed using FAERS data, resampling the dataset improved both the accuracy and the MCC of the training set (accuracy: 0.71–0.78, MCC: 0.56–0.76) but could not generalize better than non-resampled MOLD2 data to the test set (accuracy: 0.51 to 0.67, MCC: 0.14–0.36).

### Connectivity Map L1000 Cell Expression Modeling

Cellular RNA expression levels in the form of microarray data have been previously investigated for their ability to predict DILI with limited predictive power (Chierici et al., 2020). In the current study, the L1000 data from the Connectivity Map was used including both the measured landmark genes as well as the inferred transcriptome. We built models using each expression data to investigate which cell lines were most successful in predicting DILI. Table 4 summarizes the model results based on our training data. However, due to the limitation of each cell only providing expression response data from a subset of drugs (Figure 2) involved in the training and test data, accuracy based on test data was not meaningful. Additional processing steps for this data involved merging across the six cell lines to generate a representative signature, testing different cutoffs for the amount of highest- and lowest-ranked genes to utilize, as well as a feature selection for determining predictor genes.

**TABLE 4:** Training Performance on Independent Cell Line based Models. Each of the six cell lines with L1000 expression data were used to build predictive models of the four DILI classes. Training performance results for the best performing model for each cell type and DILI class are shown as well as the number of predictors following feature selection as described in the methods section.

DILI class	Cell type tested	ML algorithm	Predictors	AUC-ROC	Sensitivity	Specificity
DILI 1	PHH	SVM	60	0.969	0.912	0.945
	Hep G2	SVM	72	0.922	0.924	0.693
	HA1E	GLM	40	0.781	0.903	0.389
	A-375	GLM	178	0.627	0.826	0.17
	MCF7	GLM	65	0.722	0.898	0.222
	PC3	RF	315	0.589	1.000	0
DILI 3	PHH	NB	50	0.931	0.547	0.957
	Hep G2	RF	75	0.913	0.942	0.625
	HA1E	SVM	176	0.922	0.953	0.788
	A-375	SVM	3,610	0.833	0.869	0.607
	MCF7	SVM	74	0.861	0.872	0.742
	PC3	SVM	345	0.844	0.863	0.606
DILI 5	PHH	GLM	8	0.723	0.484	0.761
	Hep G2	RF	17	0.719	0.984	0.229
	HA1E	GLM	20	0.711	0.693	0.513
	A-375	GLM	24	0.724	0.786	0.561
	MCF7	RF	38	0.679	0.803	0.355
	PC3	GLM	14	0.661	0.255	0.961
DILI 6	PHH	GLM	2	0.574	0.087	0.990
	Hep G2	RF	31	0.686	0.000	1.000
	HA1E	RF	27	0.688	0.247	0.949
	A-375	GLM	16	0.619	0.181	0.945
	MCF7	RF	24	0.689	0.186	0.975
	PC3	RF	53	0.724	0.159	0.986

**FIGURE 6:**
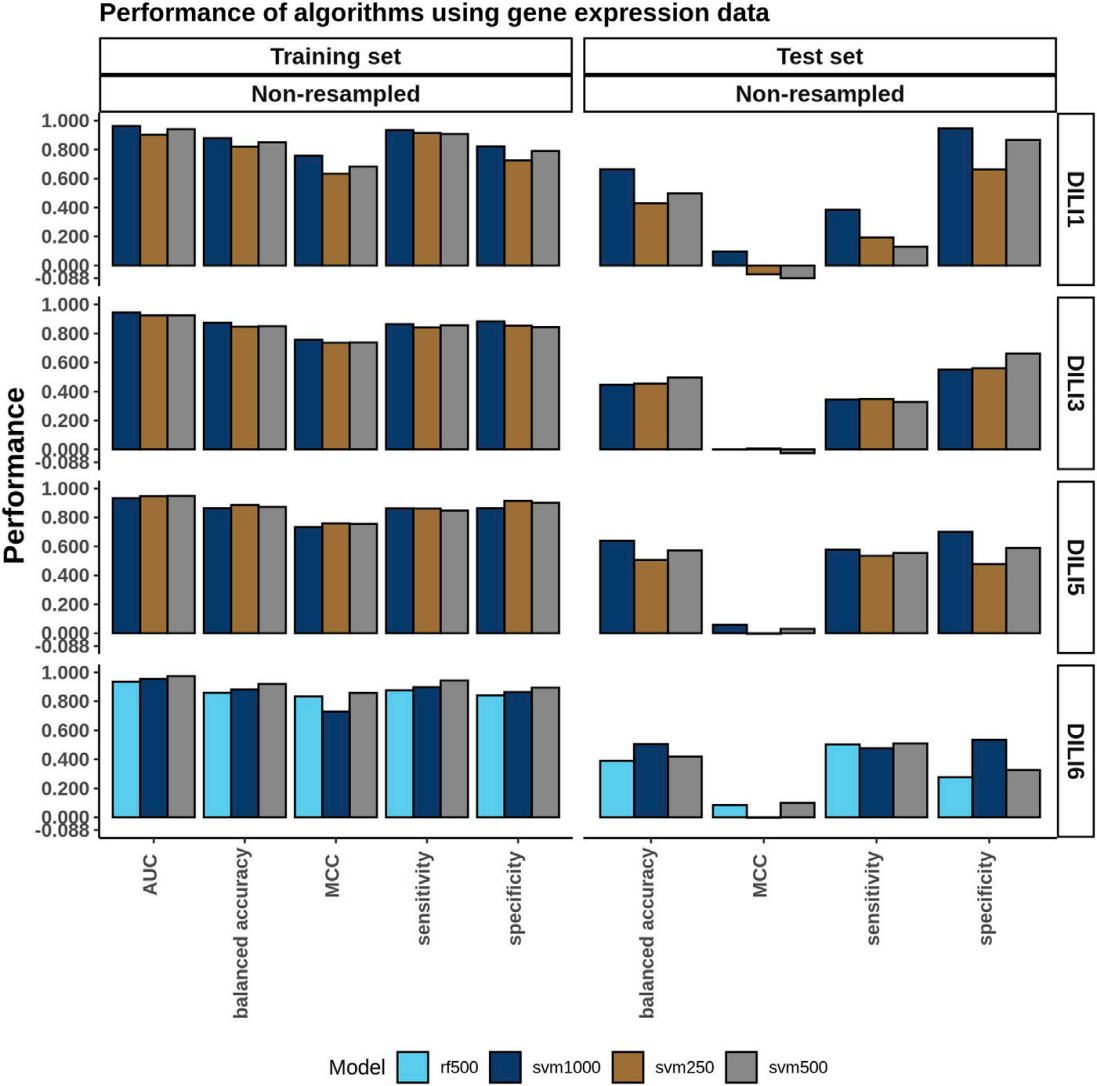
Cell Expression Model Performance. A single cell expression signature for each drug was generated using Kru-Bor merging across all cell lines in which the drug was tested as described in the methods. Following merging, feature selection using a fisher’s exact test was performed on expression signatures of the top and bottom 100, 250, 500, and 1,000 ranked genes. Models built on these predictors were evaluated and the top-performing ones, based on AUC, are shown in the training set bar graph.

The models built using the merged expression signatures with the highest AUC from the training data were evaluated on the test set. The training and test results are summarized in the bar plots in Figure 6. None of the cell expression signatures performed well when predicting DILI3, DILI5, or DILI6 with an accuracy ranging from 0.39 to 0.64 and MCC values ranging from −0.03 to 0.1. These models did have some limited success predicting DILI1 with the merged SVM 1000 model performing the best, reaching an accuracy of 0.67 but an MCC of 0.10 (Supplementary Table 1). The poor predictability of DILI3 status by these models was unexpected with the accuracy of the best model being 0.49 with 0.33 sensitivity and 0.66 specificity. The limited success in predicting DILI5 and DILI6 was expected based on the positive and negative control construction of these DILI classes, which are not reflected in the gene expression data.

### Ensemble Voting Models Performance

**FIGURE 7:**
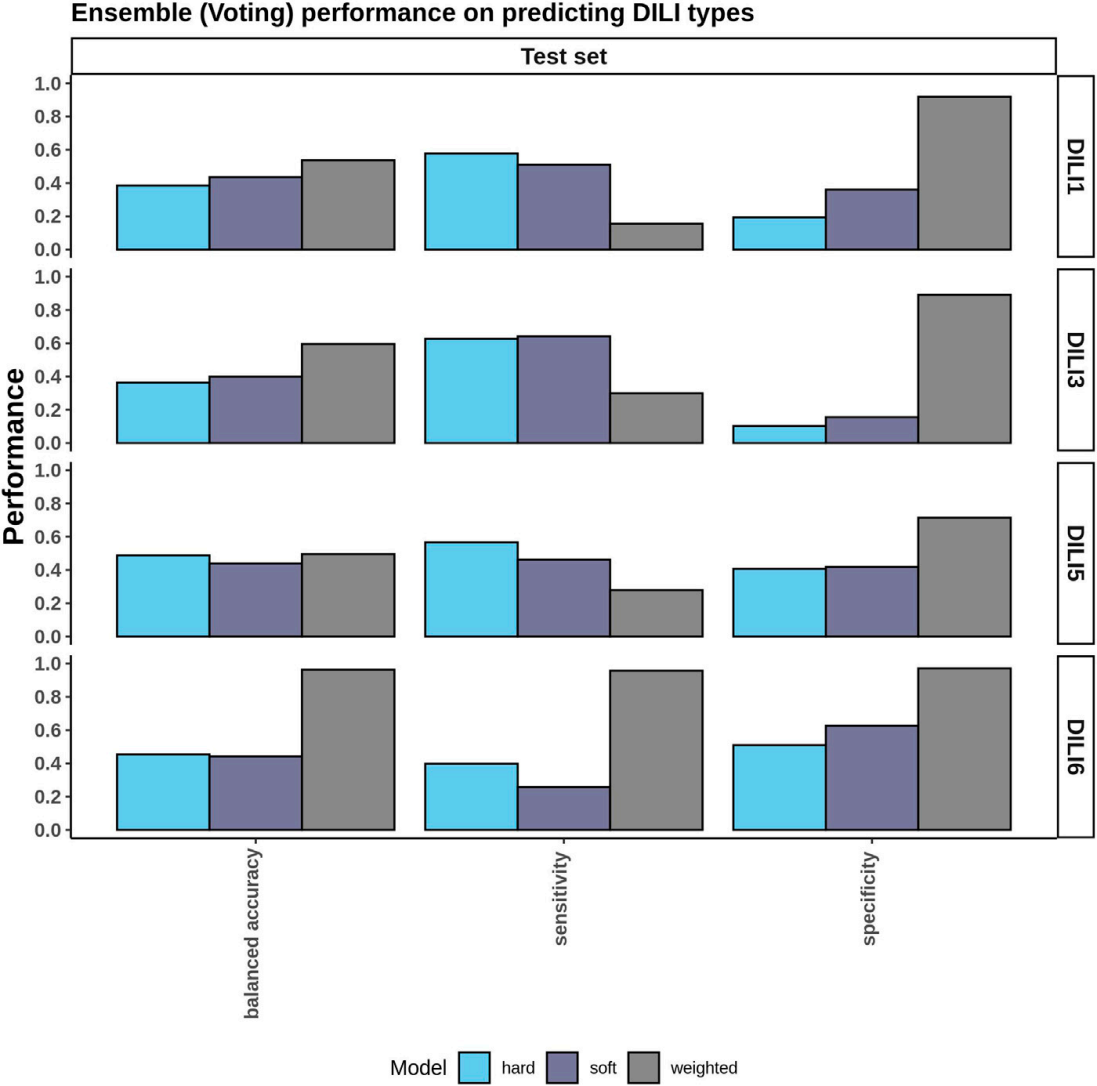
Ensemble Voting Method Performance. To incorporate the various types of data provided ensemble methods including hard, soft, and weighted voting were tested using the top three performing models for each DILI type.

Since the top three individual models did not perform well on the test set (Supplementary Table 1), we asked if aggregating the top three models in an ensemble approach could improve the accuracy. To test this, we applied three ensemble voting methods namely soft voting, hard voting, and weighted voting. Hard voting gave accuracies of 0.39 and 0.37 on DILI1 and DILI3, respectively, while soft voting gave an accuracy of 0.44 and 0.40 for DILI1 andDILI3, respectively (Figure 7). Soft voting slightly improved the accuracy of these models most likely because it considers membership probabilities rather than predicted class labels. We observed that weighted voting slightly improved the accuracy: 0.54 for DILI1 and 0.60 for DILI3. Our weighted approach considers both the probabilities and the AUC of the models and emphasizes the contribution of models with higher AUCs. Sumsion and others used similar approaches (soft and hard voting) with gene expression data resulting in decreased accuracies (Sumsion et al., 2020). Compared to their study, our approach improved the accuracies of the models. However, our method(s) does not report MCCs because we do not have access to the true positives, true negatives, false positives, and false negatives in the test data.

## Discussion

CAMDA 2020 was a collaborative challenge to establish predictive models of DILI using gene expression data as well as a combination of data from clinically reported events, drug structure, and toxicology. In our study, we evaluated the predictability of these datasets on four DILI types, namely, DILI1 (severity score ≥6), DILI3 (withdrawn, box warning, warning, and precaution), DILI5 (negative control), and DILI6 (positive control). These datasets included gene expression/perturbation data on six cell lines (PHH, HEPG2, HA1E, A375, MCF7, and PC3), concentration-response or toxicology information, 2D molecular descriptors of the drug structure, and reported adverse events. To assess the predictive abilities of these datasets, we used various traditional machine learning algorithms. For non-gene expression datasets, we corrected the imbalance issue using well-known techniques like SMOTE, ROSE, and upsampling the minority class.

While CAMDA previously approached predicting DILI, there have been significant improvements in the data provided and scopes of the challenge each year. In 2018 the challenge data only included microarray expression data from non-liver relevant cell lines on 276 compounds with a binary DILI classification. Published results from the 2018 challenge indicate limited success from both deep learning and soft voting approaches which achieved a maximum accuracy of 0.7 and MCC values < 0.2 (Chierici et al., 2020; Sumsion et al., 2020). When the CMap drug safety challenge was readministered in 2019, the data expanded to L1000 transcriptomic data on 13 cell lines and allowed participants to use external data sources such as protein-protein interactions, drug-protein targets, and chemical descriptors. The DILI classifications for this challenge also changed from binary to a most, less, ambiguous, and no-DILI concern which is in line with the FDA DILIrank dataset. Predictive model rates from multiple distinct approaches to this challenge in 2019 often yielded similar accuracy results around 0.70 (Aguirre-Plans et al., 2021; Lesiński et al., 2021; Liu et al., 2021). While it is difficult to make a direct comparison across the years of these challenges considering how the fundamental elements of predictive modeling, such as the data sources and classifications, have changed, the goal of the challenge has remained the same in modeling the risk of a drug to lead to liver injury in patients. The data structure of the challenge has also improved in each iteration attempting to expand the predictive data power as well as the data sample size to allow for more robust modeling. However, as in previous years, the highest accuracy we were able to achieve in the current study was 0.67 for DILI1 and DILI3 with the highest MCC value of 0.36. This suggests that there is still room for improvement in both model construction and developing robust predictive data, which captures the scope of DILI.

In our study, we developed models with gene expression data using individual cell lines, as well as a merging of these datasets. Each cell line dataset did not include all the drugs thereby reducing the size of the training data and making it difficult to evaluate each of them on the test set. Therefore, we merged these datasets into one expression signature across cell types. Further, we selected the 100, 250, 500, and 1,000 most upregulated and downregulated genes as an arbitrarily signature cutoff of the most perturbed genes by drug treatment. However, our approach failed to capture predictive differences between the positive and negative classes in each DILI type. Although we achieved an accuracy of 0.67 for DILI1 (on the test set), a sensitivity of 0.38 showed that our models were not learning the positive classes well enough. Usually, this problem is due to not having sufficient training examples for a particular class. In contrast, we could obtain specificity as high as 0.95, showing that the model could learn the negative classes well since there were more DILI negative drugs in the training set. Table 5 summarizes the best performances on the test set. We observed that many of these models failed to generalize to the test set i.e. they showed poor predictability on the test set (see Supplementary Table 1 for all models). Since the individual models did not perform well on the test set, we attempted ensemble (voting) methods to improve prediction accuracy. We used soft voting, hard voting, and weighted voting approaches. In weighted-voting methods, there are diverse ways through which importance can be attached to each model. Weight-based ensemble methods tend to outperform single models, and even soft voting, because in addition to the posterior probabilities churned out by the models, they take into consideration some importance or weighting factor (Mu et al., 2009). Although these methods could not improve test accuracy beyond individual models, weighted voting performed better than soft and hard voting because weighs the predicted probabilities of the test examples by the performance of each model.

**TABLE 5:** Testing Performance of Top Models. The testing result metrics from the best model built using each dataset as well as the ensemble voting model.

Dataset	DILI class	Algorithm	Test sensitivity	Test specificity	Test MCC	Test balanced accuracy
Merged expression	DILI1	SVM	0.38	0.95	0.1	0.67
	DILI3	SVM	0.33	0.66	-0.03	0.49
	DILI5	SVM	0.58	0.7	0.06	0.64
	DILI6	SVM	0.48	0.53	0	0.51
FAERS	DILI1	NNET	0.51	0.73	0.21	0.62
	DILI3	RF	0.54	0.71	0.24	0.62
	DILI5	RPART	0.51	0.57	0.08	0.54
	DILI6	RF	0.72	0.47	0.2	0.6
MOLD2	DILI1	SVMPoly	0.33	0.88	0.24	0.61
	DILI3	SVMPoly	0.55	0.8	0.36	0.67
	DILI5	SVMPoly	0.38	0.64	0.01	0.51
	DILI6	SVMPoly	0.95	0.99	0.94	0.97
TOX21	DILI1	NNET	0.3	0.82	0.12	0.56
	DILI3	GLM	0.43	0.76	0.19	0.59
	DILI5	GLM	0.3	0.75	0.06	0.53
	DILI6	QDA	0.63	0.62	0.26	0.63
Ensemble voting	DILI1	Weighted voting	0.16	0.92	0.11	0.54
	DILI3	Weighted voting	0.3	0.89	0.24	0.6
	DILI5	Weighted voting	0.28	0.71	-0.01	0.5
	DILI6	Weighted voting	0.96	0.97	0.93	0.96

One challenge we had was that the training set was perhaps too small to be further split into a training and validation set. However, machine learning algorithms benefit most from having sufficient examples. For some datasets such as the gene expression datasets, we did not have access to information on all 617 drugs, which reduced the size of the training data. Besides, the training data were largely unbalanced (Table 3). For instance, for DILI1, there were 96 positive examples and 326 negative examples. This problem resulted in many of our models having low sensitivities since the positive examples were insufficient. In an attempt to address this problem, we employed resampling techniques (SMOTE, ROSE, and upsampling minority classes) to balance the datasets. However, it was clear that models built using balanced (resampled) data were overfitting the training set. A possible reason for this was that due to our resampling approach, some training examples were also used in the validation stage during cross-validation. In addition, due to having blinded datasets, we could not explore how the features were influencing the models.

In summary, our study suggests that currently available data, including mRNA quantification, molecular descriptors, clinically reported events, and toxicology profiles, may be inadequate to capture important information enough to separate DILI classes in real-world scenarios. Also, larger datasets may be needed to encourage the application of deep learning algorithms which typically do better with bigger data. We also suggest an additional focus or challenge to predict biomarkers specific for DILI using various–omics data, for instance, single-cell data and metabolomics signatures.

## Data Availability

Data are available for download as provided by the CAMDA organizers at http://camda2020.bioinf.jku.at/doku.php/contest_dataset. The full processing code of the data for the results obtained in this article can be found at https://github.com/hurlab/CAMDA-Challenge-2020-Drug-Induced-Liver-Injury.
